# Theobromine Inhibits Uric Acid Crystallization. A Potential Application in the Treatment of Uric Acid Nephrolithiasis

**DOI:** 10.1371/journal.pone.0111184

**Published:** 2014-10-21

**Authors:** Felix Grases, Adrian Rodriguez, Antonia Costa-Bauza

**Affiliations:** Laboratory of Renal Lithiasis Research, University Institute of Health Sciences Research (IUNICS-IdISPa) and Faculty of Science of the University of the Balearic Islands, Palma, Spain; IPK, Germany

## Abstract

**Purpose:**

To assess the capacity of methylxanthines (caffeine, theophylline, theobromine and paraxanthine) to inhibit uric acid crystallization, and to evaluate their potential application in the treatment of uric acid nephrolithiasis.

**Materials and Methods:**

The ability of methylxathines to inhibit uric acid nucleation was assayed turbidimetrically. Crystal morphology and its modification due to the effect of theobromine were evaluated by scanning electron microscopy (SEM). The ability of theobromine to inhibit uric acid crystal growth on calculi fragments resulting from extracorporeal shock wave lithotripsy (ESWL) was evaluated using a flow system.

**Results:**

The turbidimetric assay showed that among the studied methylxanthines, theobromine could markedly inhibit uric acid nucleation. SEM images showed that the presence of theobromine resulted in thinner uric acid crystals. Furthermore, in a flow system theobromine blocked the regrowth of post-ESWL uric acid calculi fragments.

**Conclusions:**

Theobromine, a natural dimethylxanthine present in high amounts in cocoa, acts as an inhibitor of nucleation and crystal growth of uric acid. Therefore, theobromine may be clinically useful in the treatment of uric acid nephrolithiasis.

## Introduction

Renal lithiasis is a highly prevalent condition, currently affecting about 10% of the worldwide population [1] and estimated to affect 30% by 2050 [2]. Since most renal calculi consist of calcium oxalate, some calcium oxalate crystallization inhibitors with medical application are well known, such as magnesium, citrate and phytate [3]–[7]. Other renal calculi consist of uric acid, but, except for one in vitro study of some glycosaminoglycans and saponins [8], no uric acid crystallization inhibitors have been described to date.

Uric acid is the final product of purine catabolism in humans. In most other mammals, such as rats and dogs, uric acid is further degraded to allantoin by the enzyme uricase [9]. In humans, a high level of urate in blood is a pathophysiological condition, which, in patients with gout, can result in the formation of monosodium urate monohydrate crystals in the synovial fluid [10].

Uric acid nephrolithiasis accounts for 7–10% of kidney stones [11]–[15]. This frequency varies with age and gender, affecting men more frequently than women, and older individuals more frequently than younger persons [16], [17]. The frequency also varies with geographic localization, with uric acid nephrolithiasis affecting 1% of patients with kidney stones in India, 4% in Sweden and Turkey and 17% in Germany [18]–[21]. The metabolic abnormality most frequently associated with uric acid nephrolithiasis is low urinary pH, followed by hyperuricosuria and low diuresis [22]–[25]. Furthermore, uric acid can induce calcium oxalate monohydrate nephrolithiasis through a heterogeneous nucleation mechanism [26], [27]. Due to the lack of uric acid crystallization inhibitors, the treatment of patients prone to the formation of uric acid stones is based on urine alkalinization, and the administration of allopurinol to patients with hyperuricemia.

**Figure 1:**
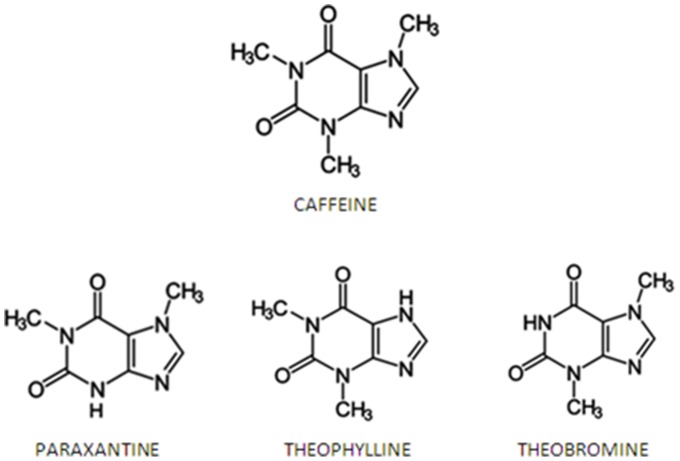
Chemical structure of methylxanthines: caffeine, theobromine, theophylline and paraxanthine. Caffeine is the 1, 3, 7-trimethylxanthine. The other three compounds are dimethylxanthines, which differ in the position of the two methyl groups.

Theobromine is a dimethylxanthine present in high amounts in chocolate and cocoa [28]. Theobromine has been less well studied than other natural methylxanthines (*figure 1*) because it stimulates the central nervous system in a lesser degree [29]. Nevertheless, theobromine consumption has health benefits, including protection of the enamel surface [30] and cough suppression [31]. Furthermore, theobromine has been shown to increase plasma HDL cholesterol and decrease plasma LDL cholesterol concentrations, conferring cardiovascular protection and reducing the risk of coronary heart disease [32], [33].

Studies in healthy volunteers showed that 50% of administered theobromine is recovered in urine after 8–12 h, and 100% is recovered after three days, suggesting that this compound is completely or almost completely absorbed [34]. The primary metabolites of theobromine were 3-methylxantine, 7-methylxantine, 7-methyluric acid and 3,7-dimethyluric acid, with 18–21% remaining unchanged [35], [36].

The aim of the present work is to study the inhibitory effect of theobromine on uric acid crystallization in synthetic urine, using different in vitro models. Theobromine concentrations used in the present study were selected according to its normal levels in urine after consumption of theobromine.

## Materials and Methods

### Reagents and solutions

Uric acid, theobromine, theophylline, caffeine and paraxanthine were purchased from Sigma-Aldrich (St. Louis, MO, USA). Synthetic urine components were obtained from Panreac (Montcada i Reixac, Barcelona, Spain). Chemicals of analytical reagent-grade purity were dissolved in ultra-pure deionized water from a Milli-Q system and filtered through 0.45 µm pore filters before use. Uric acid stock solution was prepared daily by dissolving 1 g uric acid in 0.5 L of water with 1 M NaOH addition. Synthetic urine was prepared by mixing equal volumes of A and B solutions (*Table 1*), neither of which contained calcium or oxalate, thus preventing the crystallization of calcium oxalate. The pH of both solutions was adjusted depending on the experiment.

**Table 1:** Composition of synthetic urine.*

Solution A (mM)		Solution B (mM)	
Na_2_SO_4_ · 10H_2_O	19.34	NaH_2_PO_4_ · 2H_2_O	15.45
MgSO_4_ · 7H_2_O	5.93	Na_2_HPO_4_ · 12H_2_O	15.64
NH_4_Cl	86.73	NaCl	223.08
KCl	162.60		

* Synthetic urine was obtained by mixing equal volumes of solutions A and B.

### Turbidimetric assay

Uric acid crystal formation in synthetic urine and the effects of potential crystallization inhibitors were assessed using a kinetic turbidimetric system, consisting of a photometer (Metrohm 662) equipped with a fiber-optic light-guide measuring cell with an attached light path 2×10 mm reflector and monochromatic light (550 nm). Crystallization was assessed at constant temperature (37°C) with magnetic stirring.

Synthetic urine solutions were adjusted to pH 5.40, and 2 g/L uric acid solution was adjusted to pH 10.70. Synthetic urine (160 mL) was added to a crystallization flask, followed by 40 mL uric acid solution. When the resulting solution reached a temperature of 37°C, 1.4–1.6 mL HCl was added to achieve the desired supersaturation of uric acid (pH 4.40–4.70), and the timer was switched on. These pH values were selected in order to have a short crystallization time (6–10 minutes without inhibitor). The pH of the final solution was measured at the beginning of each experiment. The absorbance of the solution (550 nm) was measured every two minutes, until the end of the kinetic assay.

Caffeine, paraxanthine and theophylline were assayed at 40 mg/L, and theobromine at concentrations of 10, 20 and 40 mg/L, each at three different pHs (4.40, 4.50 and 4.65). Each experiment was repeated twice in order to assess its reproducibility. In some cases, the crystals formed during the turbidimetric assay were filtered through a 0.45 µm pore filter, dried in a desiccator, and examined by scanning electron microscopy.

### Post-extracorporeal shock wave lithotripsy regrowth fragments

Fifteen post extracorporeal shock wave lithotripsy (ESWL) fragments of uric acid calculi, of similar weight and morphology, were obtained from a single patient. This study was a retrospective evaluation of a clinical patient sample. The volunteer had provided written informed consent for its clinical samples to be used in scientific studies. This study was approved by the “Comitè d'Ètica de la Recerca (CER)” of the University of the Balearic Islands.

**Figure 2:**
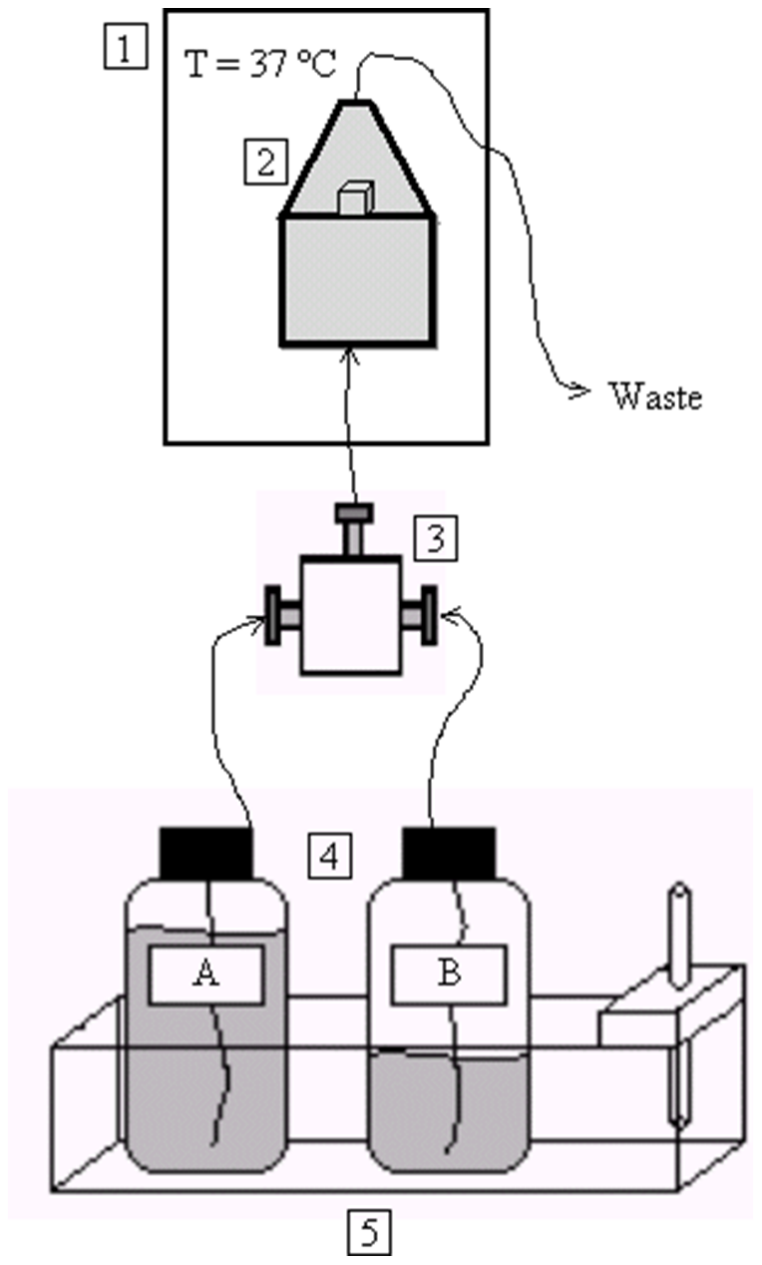
Diagram of the experimental flow device used to assess uric acid renal calculi regrowth. The device shown here is used to study the regrowth of post ESWL fragments of uric acid calculi. 1. Temperature-controlled chamber; 2. Chamber containing post-ESWL uric acid calculi fragments solutions; 3. Three-way T mixing chamber for solutions A and B; 4. Uric acid (B) and synthetic urine (A) solutions; 5. Temperature controller.

The fragments were not pre-treated and were placed in a hermetic flow chamber. This chamber was placed in a larger, temperature-controlled (37°C) chamber, and incubated for 48 h. The experimental flow device used (*Figure 2*) was similar to that used to study the regrowth of calcium oxalate dihydrate (COD) crystals on COD calculi [37], [38] and calcium oxalate monohydrate crystals on uric acid calculi [27]. Using a multichannel peristaltic pump, freshly prepared synthetic urine (pH 3.00), with or without theobromine (10 or 20 mg/L), was introduced into the flow chamber at a rate of 600 mL/day. Using a second pump, 2 g/L uric acid (pH 10.70) was introduced into the flow chamber at a rate of 150 mL/day. Both solutions were at 37°C and were mixed in a three-way T mixing chamber. Thus, 750 mL of synthetic urine every 24 hours passed through the chamber, which is approximately the volume of urine that passed through one kidney per day. We measured the pH of the waste solution, after passing through the stone fragments, to check that it was the same pH value in all the experiments.

The calculi were dried in a desiccator before and after each experiment until they reached constant weight, as determined using a precision balance, and fragment growth was evaluated by measuring the difference in weight. Mean growth rates were determined, and fragment growth was standardized by calculating the relative mass increase, thus avoiding the effects of surface area on growth rate.

The morphology of the growing calculi was assessed by scanning electron microscopy.

## Results

**Figure 3:**
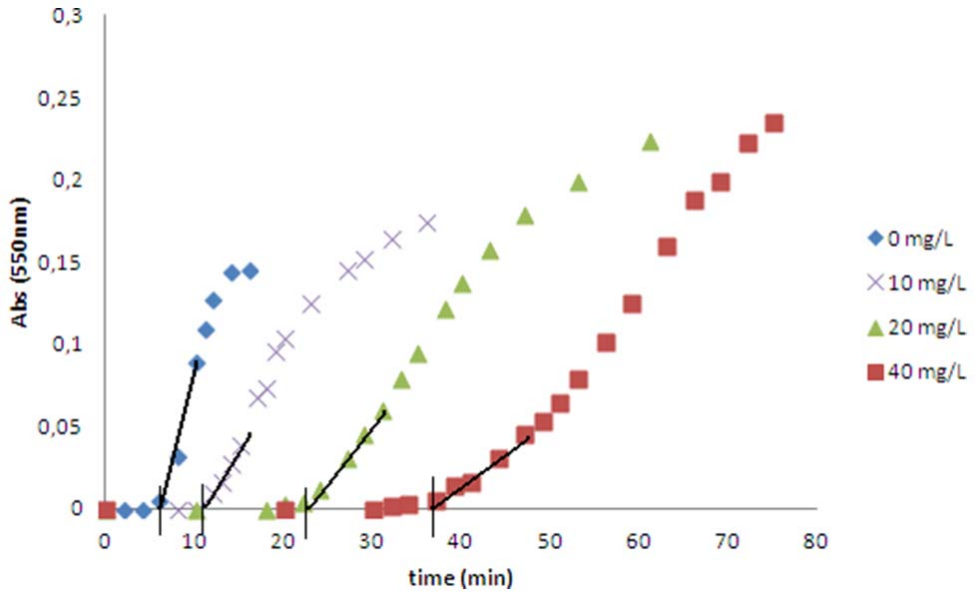
Kinetic curves for uric acid crystallization. The curves were recorded for uric acid crystallization using a turbidimeter in synthetic urine, without and with 10, 20 and 40 mg/L theobromine at pH 4.65. The calculation of induction time is represented by a solid black line.

**Figure 4:**
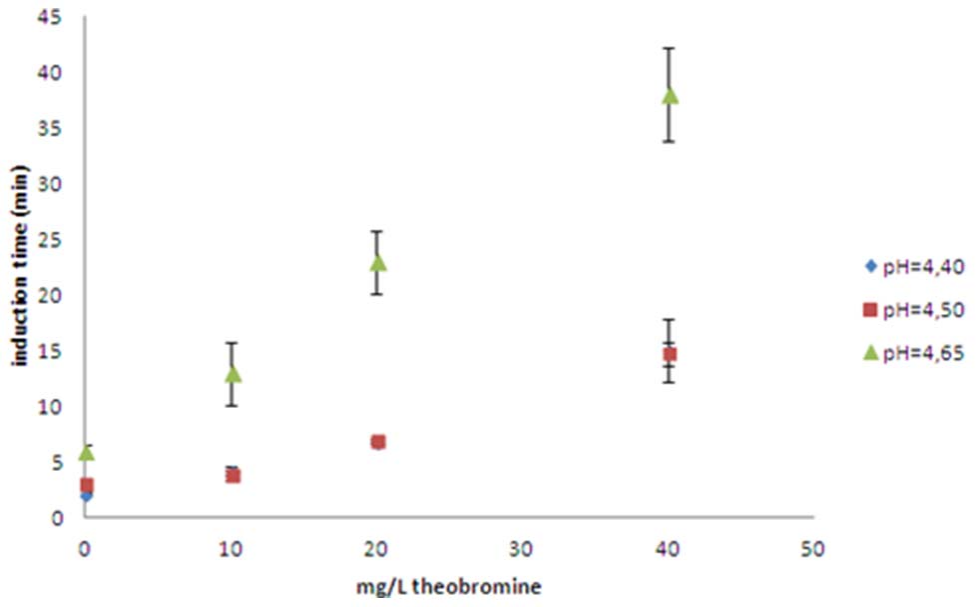
Uric acid crystallization induction times (mean ± SD) in synthetic urine. The induction times were calculated from the crystallization curves, recorded turbidimetrically, at four theobromine concentrations (0, 10, 20 and 40 mg/L), and three pH values (4.40, 4.50 and 4.65). The results are expressed as mean of duplicates and standard deviation.

**Figure 5:**
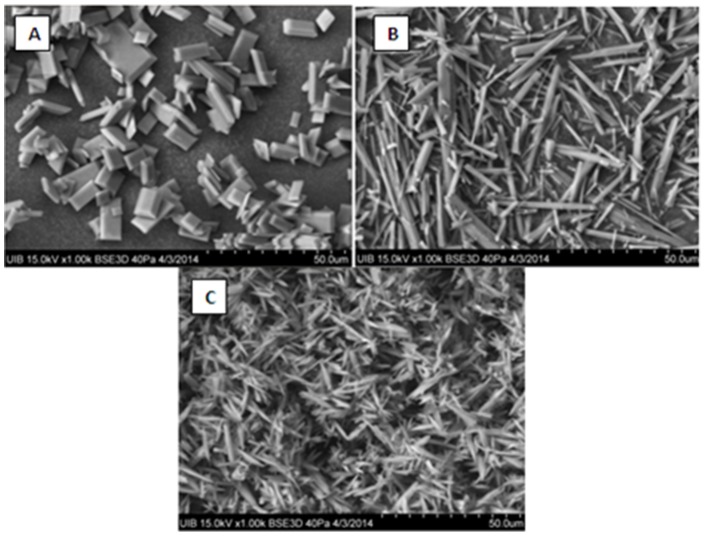
Scanning electron microscopy of uric acid crystals. After finishing the turbidimetrical assay, some of the solutions were filtered through a 45 µm filter, and uric acid crystals were observed by scanning electron microscopy. These images correspond to uric acid crystals formed at pH = 4.65, in the absence (A) and presence of 20 mg/L (B) and 40 mg/L (C) theobromine.

*Figure 3* shows the kinetic curves for the formation of uric acid crystals at three theobromine concentrations, with the induction time for each concentration calculated as shown. Caffeine, theophylline and paraxanthine at concentrations of 40 mg/L did not alter the induction time. Thus, the effects of theobromine on uric acid crystallization were assessed at different pHs and different theobromine concentrations. Induction time increased as pH and theobromine concentration increased (*Figure 4*). Scanning electron microscopy of crystals formed at pH 4.65 showed that theobromine inhibited uric acid crystallization in a concentration-dependent manner (*Figure 5*). Moreover, the uric acid crystals were thinner and longer in the presence than in the absence of theobromine. We selected the crystals formed at pH 4.65 because, as the induction time was longer, the crystals were formed slower, leading to bigger crystals, allowing us to see better in which faces the inhibitor acts.

**Figure 6:**
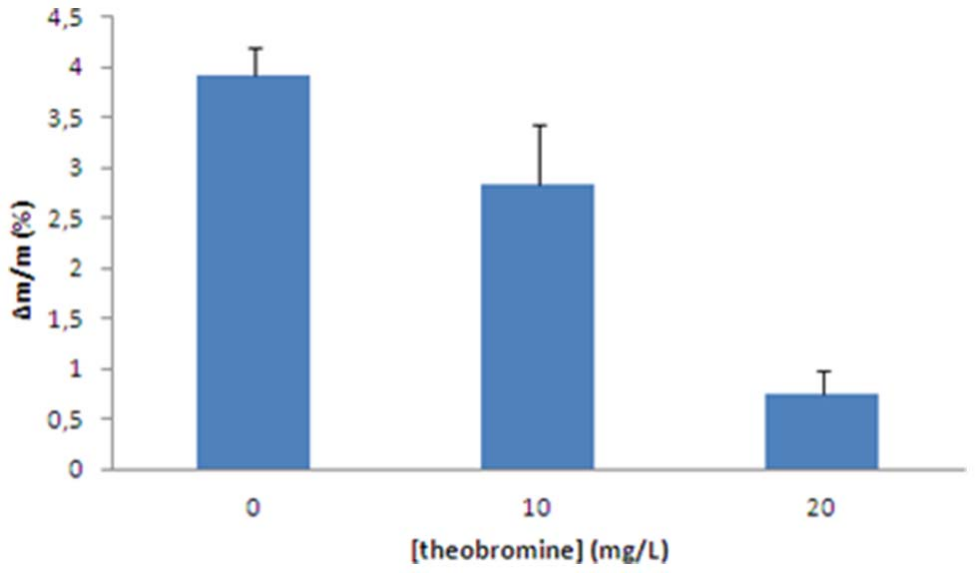
Increased relative weight of post-ESWL uric acid calculi fragments. The fragments were placed into a temperature-controlled flow chamber and incubated for 48 h. A constant flow of 400 mg/L uric acid in synthetic urine, with 0, 10 and 20 mg/L theobromine, passed through the flow chamber containing the calculus fragment. A total volume of 750 mL synthetic urine passed every 24 hours, which is approximately the volume that passes through one kidney per day. The calculi fragments were weight before and after the experiment, and the relative weight increase was calculated for each fragment. In this figure, the relative weight variation are represented as a mean ± SEM for 5 calculi fragments.

**Figure 7:**
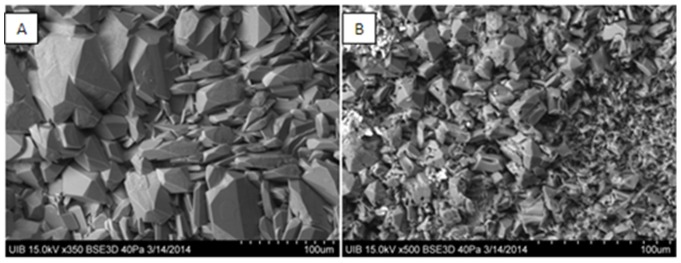
Scanning electron microscopy of uric acid crystal formation on a post-ESWL uric acid calculus fragment. The fragments were incubated in a temperature-controlled chamber during 48 h with a constant flow of 400 mg/L uric acid in synthetic urine, without (A) and with (B) 20 mg/L theobromine.

*Figure 6* shows the effects of theobromine on the regrowth of post-ESWL uric acid calculi when incubated in a flow chamber for 48 h with 400 mg/L of uric acid in synthetic urine. Theobromine showed a concentration dependent inhibition of calculi regrowth. The pH of the waste solution was around 5.50, which is a common urinary pH value in uric acid stone former patients. Moreover, scanning electron microscopy of these calculi fragments showed that the new uric acid crystals were smaller in the presence than in the absence of theobromine (*Figure 7*).

## Discussion

The results presented here indicate that, in vitro, theobromine can markedly inhibit uric acid crystallization. Turbidimetric experiments showed that theobromine can inhibit uric acid nucleation, since the delay in induction time was dependent on theobromine concentration. In contrast, caffeine, theophylline and paraxanthine had no effects. This was surprising, inasmuch as their structures are very similar. However, crystallization inhibitors are extremely selective, such that slight modifications in chemical structure can render inhibitors ineffective.

Theobromine concentrations used in this study were similar to those observed in urine after consumption of theobromine [39]–[41]. Thus, following the administration of 300 mg theobromine, 60 mg will be excreted unchanged in the urine, resulting in urinary theobromine concentrations similar to those used in this study.

The theobromine-induced delay in induction time was higher at higher pH, due to the decreased supersaturation of uric acid at increased pH. Thus, the effect of theobromine at pHs between 5 and 5.5 (common urinary pHs values in uric acid stone former patients) will be even more important, since the supersaturation of uric acid decreases when urinary pH increases. The increased effect of theobromine at higher pH suggests that urinary alkalinization combined with theobromine may be effective clinically.

**Figure 8:**
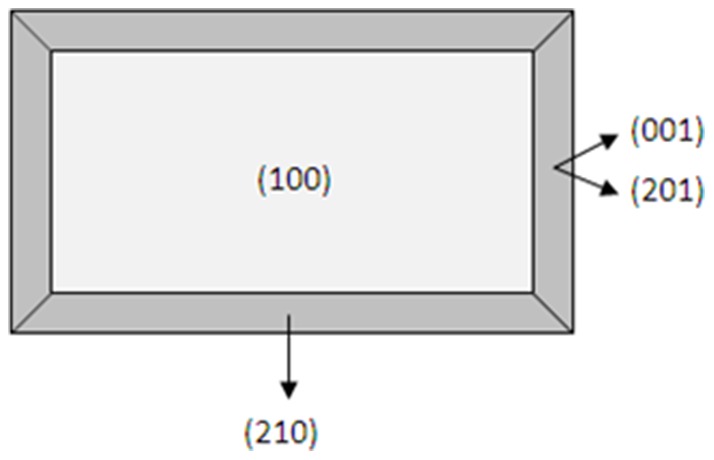
Morphology of an anhydrous uric acid crystal with its crystallographic planes. This morphology is the same that can be observed at the scanning electron microscopy when in vitro uric acid crystals are formed in the absence of admixtures (figure 5A). Due to the morphology of the uric acid crystals formed in the presence of theobromine in synthetic urine (figures 5B and 5C), we can conclude that the inhibited face is (210), which results in thinner and longer uric acid crystals.

Theobromine also altered the morphology of uric acid crystals (*figure 8*), making them longer and thinner. Theobromine may inhibit growth at only one of the faces of the crystal, (210), but not at the others, (001) and (201). As uric acid and theobromine molecules have very similar structural patterns, it could be admitted that theobromine can substitute uric acid molecules in the corresponding uric acid crystals. The incorporation of this molecule to the uric acid crystal lattice will modify the structure of some layers that, as consequence, will increase their energy, decreasing thus their growth rate. So, it is very likely to think that the face (210) is affected by the incorporation of theobromine to the uric acid crystal lattice.

Using post-ESWL uric acid fragments, we found that theobromine also inhibits the regrowth of these calculi in a concentration dependent manner. Furthermore, the crystals formed on the surface of the uric acid were smaller in the presence than in the absence of theobromine. Thus, theobromine may be useful clinically in preventing the regrowth of uric acid calculi fragments after ESWL.

The experiments described in this paper were all performed in synthetic urine. The morphology of the crystals showed in this paper may change in real urine, due to the presence of other substances in urine, such as proteins.

In conclusion, theobromine acts as a uric acid crystallization inhibitor, with high clinical potential in the treatment and prevention of uric acid nephrolithiasis. Clinical trials are necessary to assess its inhibitory capacity in vivo.

## References

[1] Ramello A, Vitale C, Marangella M (2000) Epidemiology of nephrolithiasis. J nephrol 13: S65–S70. 11132032

[2] Brikowski TH, Lotan Y, Pearle MS (2008) Climate-related increase in the prevalence of urolithiasis in the United States. Proc Natl Acad Sci U S A 105: 9841–9846. 18626008 10.1073/pnas.0709652105 PMC2474527

[3] Li MK, Blacklock NJ, Garside J (1985) Effects of magnesium on calcium oxalate crystallization. J Urol 133: 123–125. 3964871

[4] Massey L (2005) Magnesium therapy for nephrolithiasis. Magnes Res 18: 123–126. 16100850

[5] Pak CY (1991) Citrate and renal calculi: new insights and future directions. Am J Kidney Dis 17: 420–425. 2008910 10.1016/s0272-6386(12)80635-4

[6] Khan SR, Kok DJ (2004) Modulators of urinary stone formation. Front Biosci 9: 1450–1482. 14977559 10.2741/1347

[7] Grases F, Isern B, Sanchis P, Perello J, Torres JJ, et al (2007) Phytate acts as an inhibitor in formation of renal calculi. Front Biosci 12: 2580–2587. 17127264 10.2741/2256

[8] Grases F, Ramis M, Villacampa AI, Costa-Bauza A (1999) Uric acid urolithiasis and crystallization inhibitors. Urol Int 62: 201–204. 10567882 10.1159/000030395

[9] Wu X, Wakamiya M, Vaishnav S, Geske R, Montgomery C Jr, et al (1994) Hyperuricemia and urate nephropathy in urate oxidase-deficient mice. Proc Natl Acad Sci U S A 91: 742–746. 8290593 10.1073/pnas.91.2.742 PMC43025

[10] Becker MA (1988) Clinical aspects of monosodium urate monohydrate crystal deposition disease (gout). Rheum Dis Clin North Am 14: 377–394. 3051156

[11] Gutman AB, Yu TF (1968) Uric acid nephrolithiasis. Am J Med 45: 756–779. 4879835 10.1016/0002-9343(68)90209-x

[12] Mandel NS, Mandel GS (1989) Urinary tract stone disease in the United States veteran population. I. Geographical frequency of occurrence. J Urol 142: 1513–1515. 2585626 10.1016/s0022-5347(17)39144-9

[13] Mandel NS, Mandel GS (1989) Urinary tract stone disease in the United States veteran population. II. Geographical analysis of variations in composition. J Urol 142: 1516–1521. 2585627 10.1016/s0022-5347(17)39145-0

[14] Gault MH, Chafe L (2000) Relationship of frequency, age, sex, stone weight and composition in 15,624 stones: comparison of resutls for 1980 to 1983 and 1995 to 1998. J Urol 164: 302–307. 10893570

[15] Grases F, Conte A, March JG, Genestar C, Costa-Bauza A, et al (1994) Epidemiology of urinary stone disease in the Balearic Islands Community. Int Urol Nephrol 26: 145–150. 8034422 10.1007/BF02768277

[16] Gentle DL, Stoller ML, Bruce JE, Leslie SW (1997) Geriatric urolithiasis. J Urol 158: 2221–2224. 9366348 10.1016/s0022-5347(01)68203-x

[17] Costa-Bauza A, Ramis M, Montesinos V, Grases F, Conte A, et al (2007) Type of renal calculi: variation with age and sex. World J Urol 25: 415–421. 17525848 10.1007/s00345-007-0177-4

[18] Ansari MS, Gupta NP, Hemal AK, Dogra PN, Seth A, et al (2005) Spectrum of stone composition: structural analysis of 1050 upper urinary tract calculi from northern India. Int J Urol 12: 12–16. 15661049 10.1111/j.1442-2042.2004.00990.x

[19] Grenabo L, Hedelin H, Pettersson S (1985) The severity of infection stones compared to other stones in the upper urinary tract. Scand J Urol Nephrol 19: 285–289. 4089554 10.3109/00365598509180271

[20] Karabacak OR, Dilli A, Saltaş H, Yalçınkaya F, Yörükoğlu A, et al (2013) Stone Compositions in Turkey: An Analysis According to Gender and Region. Urology 82: 532–538. 23987145 10.1016/j.urology.2013.04.059

[21] Hesse A, Schneider HJ, Berg W, Hienzsch E (1975) Uric acid dihydrate as urinary calculus component. Invest Urol 12: 405–409. 1112668

[22] Bell DS (2012) Beware the low urine pH – the major cause of the increased prevalence of nephrolithiasis in the patient with type 2 diabetes. Diabetes Obes Metab 14: 299–303. 21992452 10.1111/j.1463-1326.2011.01519.x

[23] Wagner CA, Mohebbi N (2010) Urinary pH and stone formation. J Nephrol 23 Suppl 16: S165–169. 21170875

[24] Ngo TC, Assimos DG (2007) Uric Acid nephrolithiasis: recent progress and future directions. Rev Urol 9: 17–27. 17396168 PMC1831527

[25] Grases F, Costa-Bauza A, Gomila I, Ramis M, Garcia-Raja A, et al (2012) Urinary pH and renal lithiasis. Urol Res 40: 41–46. 21594588 10.1007/s00240-011-0389-3

[26] Grases F, Sanchis P, Perello J, Costa-Bauza A (2006) Role of uric acid in different types of calcium oxalate renal calculi. Int J Urol 13: 252–256. 16643619 10.1111/j.1442-2042.2006.01262.x

[27] Grases F, Sanchis P, Isern B, Perello J, Costa-Bauza A (2007) Uric acid as inducer of calcium oxalate crystal development. Scand J Urol Nephrol 41: 26–31. 17366099 10.1080/00365590600831571

[28] Craig WJ, Nguyen TT (2006) Caffeine and Theobromine Levels in Cocoa and Carob Products. J Food Sci 49: 302–303.

[29] Gates S, Miners JO (1999) Cytochrome P450 isoform selectivity in human hepatic theobromine metabolism. Br J Clin Pharmacol 47: 299–305. 10215755 10.1046/j.1365-2125.1999.00890.x PMC2014222

[30] Kargul B, Ozcan M, Peker S, Nakamoto T, Simmons WB, et al (2012) Evaluation of human enamel surfaces treated with theobromine: a pilot study. Oral Health Prev Dent 10: 275–282. 23094271

[31] Halfdanarson TR, Jatoi A (2007) Chocolate as a cough suppressant: rationale and justification for an upcoming clinical trial. Support Cancer Ther 4: 119–122. 18632476 10.3816/SCT.2007.n.006

[32] Khan N, Monagas M, Andres-Lacueva C, Casas R, Urpi-Sarda M, et al (2012) Regular consumption of cocoa powder with milk increases HDL cholesterol and reduces oxidized LDL levels in subjects at high-risk of cardiovascular disease. Nutr Metab Cardiovasc Dis 22: 1046–1053. 21550218 10.1016/j.numecd.2011.02.001

[33] Neufingerl N, Zebregs YE, Schuring EA, Trautwein EA (2013) Effect of cocoa and theobromine consumption on serum HDL-cholesterol concentrations: a randomized controlled trial. Am J Clin Nutr 97: 1201–1209. 23595874 10.3945/ajcn.112.047373

[34] Tarka SM Jr, Arnaud MJ, Dvorchik BH, Vesell ES (1983) Theobromine kinetics and metabolic disposition. Clin Pharmacol Ther 34: 546–555. 6617078 10.1038/clpt.1983.212

[35] Rodopoulos N, Hojvall L, Norman A (1996) Elimination of theobromine metabolites in healthy adults. Scand J Clin Lab Invest 56: 373–383. 8837245 10.3109/00365519609090590

[36] Shively CA, Tarka SM Jr, Arnaud MJ, Dvorchik BH, Passananti GT, et al (1985) High levels of methylxanthines in chocolate do not alter theobromine disposition. Clin Pharmacol Ther 37: 415–424. 3979003 10.1038/clpt.1985.65

[37] Chow K, Dixon J, Gilpin S, Kavanagh JP, Rao PN (2004) Citrate inhibits growth of residual fragments in an in vitro model of calcium oxalate renal stones. Kidney Int 65: 1724–1730. 15086911 10.1111/j.1523-1755.2004.00566.x

[38] Costa-Bauza A, Perello J, Isern B, Sanchis P, Grases F (2006) Factors affecting calcium oxalate dihydrate fragmented calculi regrowth. BMC Urol 6: 16–22. 16822299 10.1186/1471-2490-6-16 PMC1526447

[39] Zambonin CG, Aresta A, Palmisano F (2004) Determination of methylxanthines in urine by liquid chromatography with diode array UV detection. J Pharm Biomed Anal 36: 621–624. 15522539 10.1016/j.jpba.2004.07.010

[40] de Aragao NM, Veloso MC, Bispo MS, Ferreira SL, de Andrade JB (2005) Multivariate optimisation of the experimental conditions for determination of three methylxanthines by reversed-phase high-performance liquid chromatography. Talanta 67: 1007–1013. 18970272 10.1016/j.talanta.2005.04.066

[41] Ptolemy AS, Tzioumis E, Thomke A, Rifai S, Kellogg M (2010) Quantification of theobromine and caffeine in saliva, plasma and urine via liquid chromatography-tandem mass spectrometry: a single analytical protocol applicable to cocoa intervention studies. J Chromatogr B Analyt Technol Biomed Life Sci 878: 409–416. 10.1016/j.jchromb.2009.12.019 20045386

